# Circulating erythropoietin concentration associates with thromboembolism in sickle cell disease

**DOI:** 10.1111/bjh.70517

**Published:** 2026-04-29

**Authors:** Xu Zhang, Binal N. Shah, Jin Han, Mehdi Nouraie, Yingze Zhang, Roberto F. Machado, Mark T. Gladwin, Santosh L. Saraf, Victor R. Gordeuk

**Affiliations:** 1 Department of Medicine, University of Illinois at Chicago, Chicago, Illinois, USA; 2 College of Pharmacy, University of Illinois at Chicago, Chicago, Illinois, USA; 3 Department of Pulmonary, Allergy, Critical Care, and Sleep Medicine, University of Pittsburgh, Pittsburgh, Pennsylvania, USA; 4 School of Medicine, University of Maryland, Baltimore, Maryland, USA

**Keywords:** erythropoietin, erythropoietin receptor, expression quantitative locus, sickle cell disease, thromboembolism

## Abstract

Elevated erythropoietin (EPO) concentration associates with thrombotic risk in hypoxic conditions, hereditary erythrocytosis and treatment of anaemia with recombinant EPO. We evaluated sickle cell disease (SCD) patients from the University of Illinois at Chicago (UIC) and the Treatment of Pulmonary Hypertension and SCD with Sildenafil Therapy (Walk-PHaSST) study and found that higher serum EPO concentration associated with increased thromboembolic risk (combined odds ratio [OR] = 1.9, *p* = 0.0029, *N* = 557). Percent haemoglobin F and haemoglobin concentration strongly correlated with EPO concentration in SCD, and the haemoglobin F locus *BCL11A* affected EPO concentration through percent haemoglobin F. In peripheral blood mononuclear cells from 159 UIC patients, we identified an expression quantitative trait locus for *EPOR* encoding EPO receptor, in which the G allele of rs322139 associated with higher *EPOR* expression (*β* = 0.055, *p* = 2.0 × 10^−5^). This G allele associated with lower EPO concentration in Walk-PHaSST (*β* = −0.23, *p* = 6.4 × 10^−5^, *N* = 327) and UIC (*β* = −0.18, *p* = 0.017, *N* = 179), but not in normal populations. The G allele of rs322139 also associated with a trend to decreased thromboembolism (combined OR = 0.64, *p* = 0.054, *N* = 665). In summary, our study indicates that higher serum EPO concentration associates with thromboembolic risk in SCD and reveals a novel role of *EPOR* expression variation in modulating EPO concentration and, possibly, thromboembolic risk in this condition.

## INTRODUCTION

Elevated erythropoietin (EPO) prevents apoptosis of colony forming unit-erythroid cells and promotes erythroblast differentiation in the bone marrow^1^ by binding to EPO receptor (EPOR) on the surface of erythroid progenitors, which leads to EPOR dimerization and initiates the Janus Kinase/Signal Transducers and Activators of Transcription cascade.^2^

EPO is produced by a distinct population of renal stromal cells^3^ located within the corticomedullary border region where there is a steep gradient of oxygen partial pressure. It is also produced by hepatocytes.^4^ EPO is primarily regulated by hypoxia inducible factor (HIF)-2, a transcription factor composed of a HIF-2α subunit and a constitutive HIF-β subunit.^5^ HIF-2α subunits are hydroxylated by oxygen- and ferrous-dependent prolyl hydroxylase domain 2 (PHD2) at specific proline residues, which enables HIF-2α interaction with Von Hippel Lindau protein for proteasomal degradation.^6^ Under hypoxia and iron deficiency, hydroxylation of HIF-2α by PHD2 is diminished, leading to decreased HIF-2α degradation, increased HIF-2 heterodimers and transcriptional activation of *EPO*.^7^ Genetic factors also regulate circulating EPO levels.^8–10^

Sickle cell disease (SCD) is caused by homozygosity for the sickle haemoglobin (HbS) mutation and common compound heterozygote genotypes of HbS with other β-globin gene mutations, including haemoglobin C (HbC) and β thalassemia (Hbβ). SCD is characterized by anaemia, chronic hypoxia and stress erythropoiesis.^11^ The increase of EPO in SCD is less than the increase in patients with other forms of anaemia but similarly low haemoglobin concentrations.^12^ This may be partially due to impaired renal function and to lower binding affinity of haemoglobin S than haemoglobin A to oxygen under low O_2_ saturation. Haemoglobin F ranges from 5% to 8% in sickle cell anaemia (haemoglobin SS) patients compared to <1% in normal adults^13^ due, in part, to stress erythropoiesis.^14^ Haemoglobin F increases even further if the patient is exposed to the myelosuppressive drug hydroxyurea (hydroxycarbamide).^14^ Haemoglobin F has high oxygen affinity^15^ and leads to increased EPO levels despite ameliorating haemolysis and anaemia.^13^ Clinical variables correlating with circulating EPO concentration in SCD have not been fully evaluated and genetic factors underlying EPO variation in SCD are not fully defined.

Elevated serum EPO concentration predicts thrombotic risk in Chuvash erythrocytosis^16^ and high altitude erythrocytosis.^17^ In randomized, controlled trials, administration of recombinant EPO to healthy male volunteers markedly enhanced endothelial activation and platelet reactivity.^18^ Treatment with recombinant erythropoietin or darbopoietin for prophylaxis or therapy of anaemia in patients with cancer or chronic kidney disease is also associated with thrombosis.^19,20^ SCD patients have elevated serum EPO concentrations and are at an increased risk for thrombosis^21^ compared to normal individuals. It is unclear, however, whether elevated serum EPO concentration predicts thrombosis in SCD. Improved understanding of EPO regulation and its impact on thrombotic risk may facilitate therapeutic innovation.^22^

## METHODS

### Patient cohorts

The Treatment of Pulmonary Hypertension and Sickle Cell Disease with Sildenafil Therapy (Walk-PHaSST) study was a screening cohort of SCD patients for their eligibly for sildenafil in a randomized clinical trial. Walk-PHaSST assembled 720 patients from 10 SCD clinical sites including University of Illinois at Chicago (UIC) between 2007 and 2009.^23^ Of these patients, 547 had serum erythropoietin concentration measured centrally (Table S1). An additional 210 UIC SCD patients who were not part of Walk-PHaSST were also studied (Table S2).^24^ The UIC cohort is a longitudinal cohort with baseline measures and ongoing follow-up. Institutional review boards of participating institutions approved the study. Blood samples were obtained at steady state defined as no vaso-occlusive episode and no emergency room visit or hospitalization in the preceding 3 weeks. Baseline clinical information was recorded at enrolment after patients signed informed consent.

### Laboratory assays

Serum EPO was measured by enzyme-linked immunosorbent assay. Serum EPO, serum ferritin and haemoglobin fractionation from the 10 sites of Walk-PHaSST were centrally assayed on baseline samples. Except for serum EPO, which was assayed in two batches by two laboratories, all clinical variables in UIC were centrally assayed. Genomic deoxyribonucleic acid (DNA) and messenger ribonucleic acid (RNA) were isolated from peripheral blood mononuclear cells (PBMCs) of the baseline samples. DNA was genotyped using Illumina Human 610-Quad single nucleotide polymorphism (SNP) array for Walk-PHaSST and Affymetrix Axiom Pan African array for UIC patients. Messenger RNA was profiled using Affymetrix HuGene 2.0 ST array.

### Genotype data

Samples with genotype call rate <95% were excluded. SNPs with genotype call rate < 95%, minor allele frequency <0.01 or autosomal SNPs deviating from Hardy–Weinberg equilibrium with *p*-value < 0.0001 were excluded. Population stratification was estimated by principal component analysis of autosomal genotypes.^25^ Outliers deviating by more than four standard deviations from the mean of the first and second principal components were removed. Identity by descent was calculated using Plink.^26^ For pairs of individuals with coefficient of relationship >0.05, one patient from each pair was randomly selected and removed from analysis. Patient genotypes were phased^27^ and imputed^28^ to African American population samples in 1000 Genomes Phase 3 data.

### Gene expression data

Array probe sequences were mapped to human genome assembly GRCh37 and annotated by Gencode version 24. Probes that interrogate multiple gene transcripts and probes that contain SNPs with minor allele frequency ≥0.05 in the 1000 Genomes dataset were removed. Probe intensities were log_2_ transformed, background corrected, quantile normalized and subtracted by the corresponding probe mean across samples. Gene expression level was summarized as mean intensity across probes. Only autosomal genes were further analysed. Batch effects of RNA labelling and array hybridization were adjusted using Combat.^29^

### Expression quantitative trait loci

Expression quantitative trait loci (eQTL) were identified in the UIC patients. Nineteen principal components were regressed out of gene expression data to optimize detection power. SNPs located <1 Mb from gene ends were tested for expression association. eQTL were identified using a permutation-based false discovery rate of 0.05.

### Statistical analyses

Statistical analyses were conducted in R version 4.2.1. Missing values in individual models were excluded from analysis. Additive mode was used in genetic associations.

## RESULTS

### Elevated serum EPO concentration associates with thromboembolism in SCD

Incidence of thromboembolism, including deep vein thrombosis and pulmonary embolism, was recorded in the UIC cohort during a median follow-up of 6.4 years.^21^ Among 204 UIC patients, 53 experienced thrombosis during follow-up, 34 with deep vein thrombosis and 23 with pulmonary embolism. Higher baseline serum EPO concentration associated with increased risk for thromboembolism with a marginal significance (odds ratio [OR] = 1.6, 95% confidence interval [CI] 0.98–2.6, *p* = 0.058, Table 1), adjusting for age, gender, haemoglobin genotype, hydroxyurea therapy, recent blood transfusion and batches of EPO measurement. The correlation was more significant among 176 UIC patients without recent blood transfusion (OR = 2.2, 95% CI 1.2–3.9, *p* = 0.0068).

Among 530 Walk-PHaSST patients, 26 had a self-reported history of pulmonary embolism.^30^ Adjusting for age, gender, haemoglobin genotype, hydroxyurea therapy and recent blood transfusion, there was a trend for serum EPO concentration to positively relate to a history of pulmonary embolism (OR = 1.6, 95% CI 0.88–2.8, *p* = 0.16, Table 1). A similar trend was observed among 381 Walk-PHaSST patients without recent blood transfusion (OR = 1.7, 95% CI 0.86–3.2, *p* = 0.17). Venous thrombosis other than pulmonary embolism was not recorded in Walk-PHaSST. The underestimation may lead to a lower statistical power in this cohort.

Meta-analysis of UIC and Walk-PHaSST resulted in an OR = 1.6, 96% CI 1.1–2.3, *p* = 0.016 for higher EPO concentration with increased risk for thromboembolism in all patients, and an OR = 1.9, 95% CI 1.3–3.0, *p* = 0.0029 in patients without recent blood transfusion. Similar results were obtained if the analysis was restricted to SS and Sβ^0^ patients (Table 1).

### Haemoglobin concentration and percent haemoglobin F are the major covariates of serum EPO concentration in SCD

**FIGURE 1:**
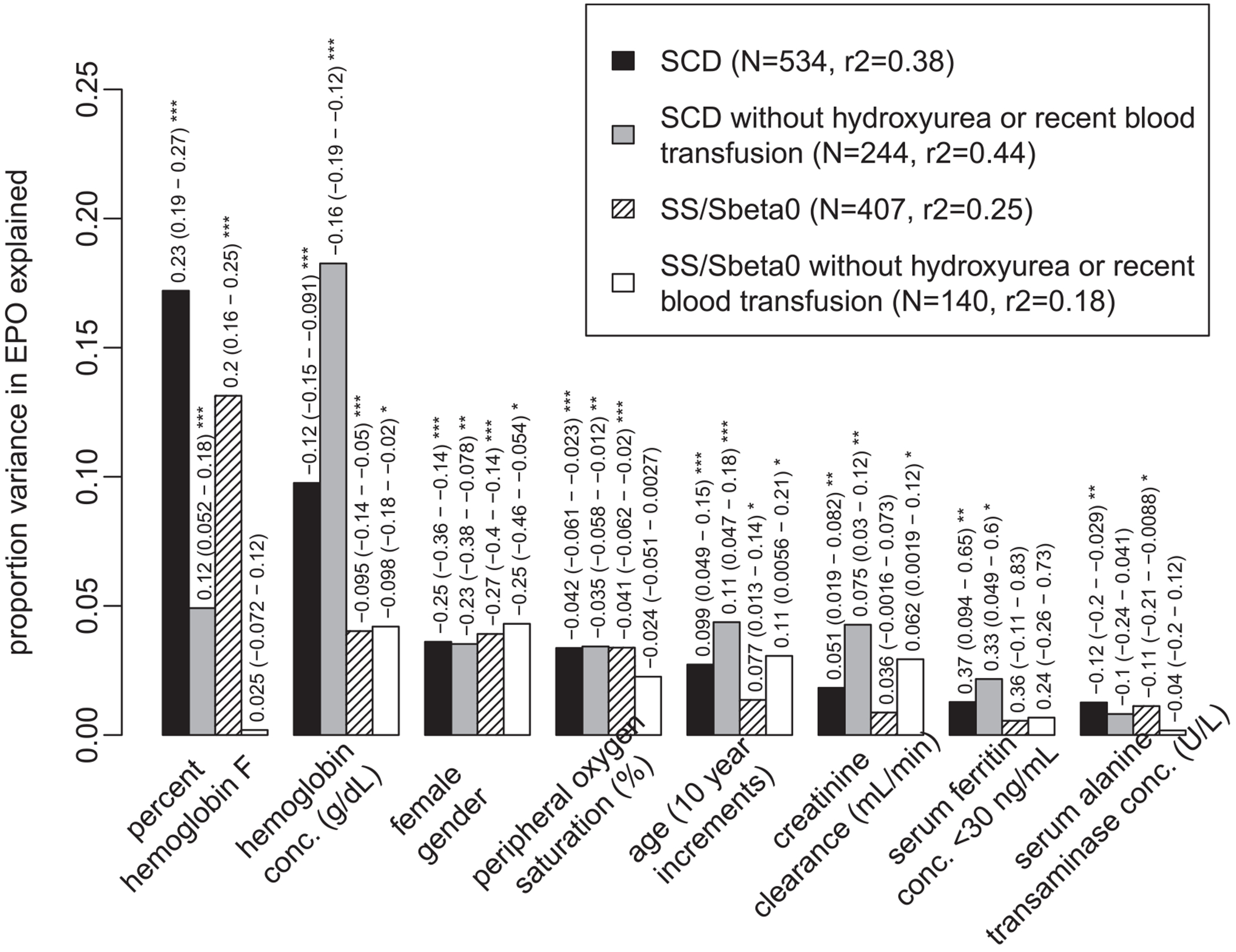
Proportion of variance in EPO explained (y-axis) by clinical variables (x-axis) in multivariate linear regression in all SCD patients from Walk-PHaSST, SCD patients without hydroxyurea therapy or recent blood transfusion, SS/Sβ^0^ patients and SS/Sβ^0^ patients without hydroxyurea therapy or recent blood transfusion. Estimated βs (95% CI) in respective models are labelled above each variable, with *p-value <0.05, **p-value <0.01 and ***p-value <0.001; *r*^2^ in the upper boxed legends shows the total variance explained by the respective multivariate model. EPO, erythropoietin; SCD, sickle cell disease.

Walk-PHaSST patients had centrally measured serum EPO concentration. We therefore examined clinical correlations in Walk-PHaSST patients, including those receiving hydroxyurea or blood transfusion within the past 2 months, and derived a model for EPO concentration in 534 Walk-PHaSST patients (Supporting Information S1). Higher percent haemoglobin F and lower haemoglobin concentration correlated with higher EPO concentration, accounting for 17% and 9.8% of EPO variation respectively (Table S3, Figure 1). Ranked in descending order of percent variance, other factors that correlated with higher EPO concentration included lower peripheral oxygen saturation (3.4%), higher creatinine clearance (1.8%) reflecting better kidney function, iron deficiency (1.3%) defined by serum ferritin concentration <30 μg/L and lower serum concentration of alanine transaminase (1.3%) reflecting better liver function. Older age and male gender also correlated with higher EPO concentration. Among patients not receiving hydroxyurea or having a recent blood transfusion, lower haemoglobin concentration was the strongest covariate of serum EPO concentration (18.3% variance) and higher haemoglobin F the second covariate (4.9% variance). Analyses stratified by treatment or demographics indicated relatively consistent correlations of these variables with EPO concentration (Table S4).

Haemoglobin concentration accounted for about 10% or more of the variance in EPO concentration if analyses included both severe and mild haemoglobin genotypes (Figure 1). This is expected as variation in haemoglobin concentration is primarily attributable to haemoglobin genotypes (Table S5). Percent haemoglobin F accounted for above 10% variance in EPO concentration if analyses included patients with recent blood transfusion or hydroxyurea therapy (Figure 1). Blood transfusion increases total haemoglobin but not haemoglobin F and therefore decreases percent haemoglobin F, whereas hydroxyurea therapy leads to increased erythrocyte haemoglobin F (Table S5). Including patients on these treatments increased overall variation in percent haemoglobin F, and thus, the proportion of EPO variation that percent haemoglobin F accounted for.

### The *BCL11A* locus affects serum EPO concentration through percent haemoglobin F

*HBS1L-MYB* is the only locus identified across genome wide association studies that consistently affects serum EPO concentration in the general population.^8–10^ SNPs in *HBS1L-MYB, BCL11A* and the β-globin locus are known to affect haemoglobin F levels in SCD.^31^ As recent blood transfusion temporarily affects percent haemoglobin F and EPO concentration, we examined the relationship of these loci with EPO concentration and assessed the mediation effect of percent haemoglobin F in 329 Walk-PHaSST patients without recent blood transfusion. In general, these loci had marginal association with EPO concentration (Table S6).

**FIGURE 2:**
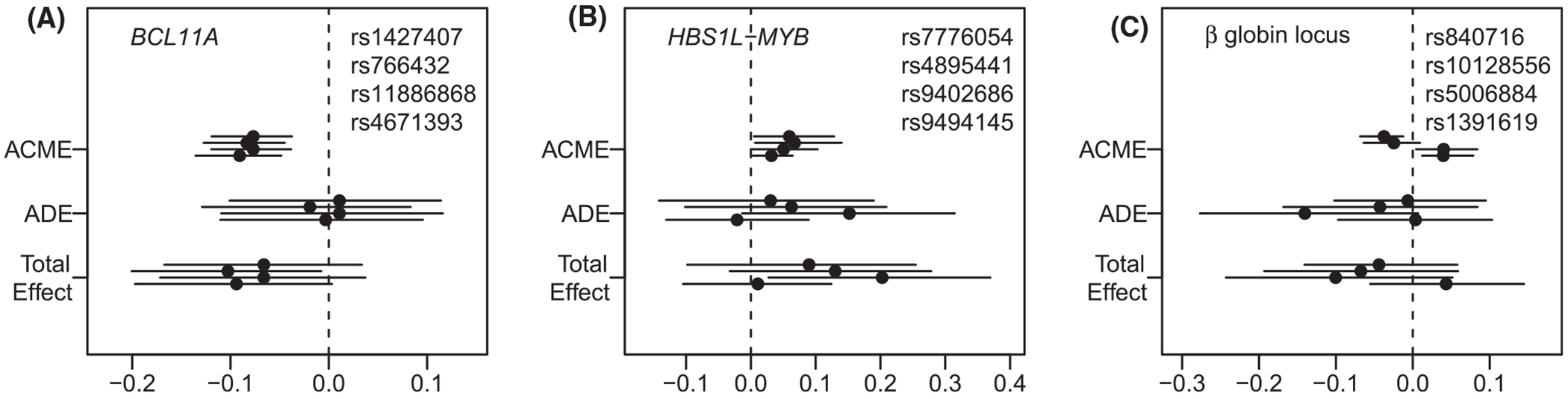
Genetic effect of SNPs in *BCL11A, HBS1l-MYB* and the *β* globin locus on serum EPO concentration was potentially mediated through percent haemoglobin F in Walk-PhaSST patients. For each SNP, the estimated average causal mediation effect (ACME) through percent haemoglobin F, average direct effect (ADE) and total effect on EPO concentration (points) and the respective 95% CI (lines) are plotted per locus. The order of the SNPs from top to bottom is the same as the order of the SNPs listed. EPO, erythropoietin; SNP, single nucleotide polymorphism.

The *BCL11A* locus strongly associated with percent haemoglobin F, adjusting for age, gender, haemoglobin concentration, hydroxyurea therapy and population stratification (Table S6). Adjusting for the same covariates, mediation analysis^32^ of the effect of the *BCL11A* locus on EPO concentration revealed a potential mediation by percent haemoglobin F, with highly significant mediation effect but non-significant direct effect (Table S6, Figure 2). The *HBS1L-MYB* and β-globin loci also exhibit modest effects on EPO concentration mediated through percent haemoglobin F.

### An eQTL of *EPOR* associates with serum EPO concentration specifically in SCD and associates with thromboembolism

Using PBMC gene expression data from 159 UIC SCD patients, we found no eQTL for *EPO* but we identified two eQTL for *EPOR* encoding EPO receptor, one of which contained two linked (*r*^2^ = 0.99) SNPs, rs322139 and rs322138. As shown in Table 2, the G allele of rs322139 associated with higher *EPOR* gene expression in these UIC PBMCs (*β* = 0.055, *p* = 2.0 × 10^−5^). Also in UIC PBMCs, *EPOR* gene expression levels correlated with a previously published erythroid gene expression signature^33^ (*β* = 0.24, *p* = 4.4 × 10^−11^), consistent with *EPOR* expression in peripheral erythroid progenitors, which make up an increased proportion of PBMCs in SCD due to heightened erythropoiesis.^33^

**FIGURE 3:**
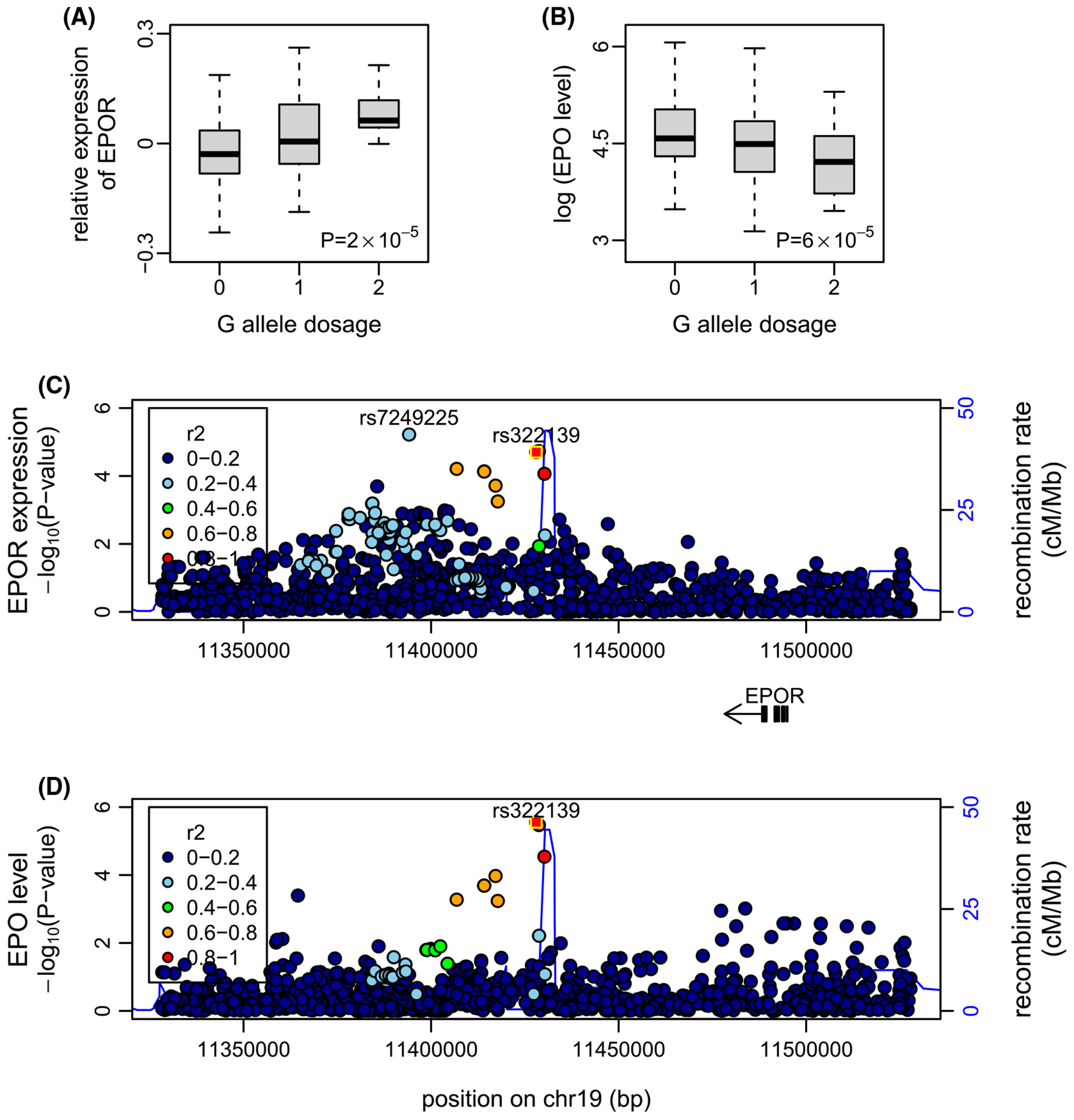
Genetic association with *EPOR* expression level in PBMCs and with circulating EPO concentration in a 200-Kb region centred at rs322139 in the *EPOR* eQTL. (A) Relative expression levels of *EPOR* are plotted according to the G allele dosage of rs322139 in UIC patients. (B) Adjusted serum EPO concentrations are plotted according to the G allele dosage in the Walk-PHaSST patients. (C) The -log_10_ *p*-values of association with *EPOR* expression at the locus. The bars under the panel denote the relative position of *EPOR* exons and the arrow denotes transcript direction. (D) The -log_10_ *p*-values of association with serum EPO concentration in the combined Walk-PHaSST and UIC patients at the locus. In C and D, the colour of points depicts the strength of linkage disequilibrium *r*^2^ between SNPs and rs322139. In C, rs7249225 belongs to the other eQTL of *EPOR*, which does not associate with EPO concentration significantly in this study. PBMC, peripheral blood mononuclear cells; SCD, sickle cell disease; UIC, University of Illinois at Chicago.

The G allele of rs322139 associated with lower serum EPO concentration in Walk-PHaSST (*β* = −0.18, *p* = 0.00029, *N* = 470) and UIC (*β* = −0.17, *p* = 0.032, *N* = 186), adjusting for age, gender, haemoglobin concentration, percent haemoglobin F, renal dysfunction defined by estimated glomerular filtration rate <60 mL/min/1.73 m^2^ and population stratification (Table 2). The associations are more significant among patients without recent blood transfusion (*β* = −0.23, *p* = 6.4 × 10^−5^, *N* = 327 in Walk-PHaSST and *β* = −0.18, *p* = 0.017, *N* = 179 in UIC). Assuming that both *EPOR* gene expression and EPO concentration have a genetic association in the region, the probability that they share a single causal variant is 0.96 using Bayesian colocalization analysis.^34^ Genetic associations with *EPOR* expression and serum EPO concentration at this *EPOR* eQTL are shown in Figure 3.

In normal populations, the rs322139 G allele does not associate with serum EPO concentration, as reported by a recent meta-analysis in individuals of European and African decent (*β* = 0.026, *p* = 0.22, *N* = 6127).^8^ That study also reports no significant heterogeneity of estimated genetic effects across individual cohorts of the meta-analysis (*p* = 0.58).^8^ These findings suggest that the association of the rs322139 G allele with lower EPO concentration is specific to SCD.

The G allele of rs322139 showed a trend of association with decreased risk for thromboembolism in UIC (OR = 0.70, *p* = 0.18, *N* = 198), adjusting for age, gender, haemoglobin genotypes, hydroxyurea therapy, recent blood transfusion and population stratification. Adjusting for the same covariates, the G allele also showed a trend of association with decreased risk for pulmonary embolism in Walk-PHaSST (OR = 0.52, *p* = 0.13, *N* = 467). Meta-analysis of the two cohorts resulted in a combined OR = 0.64, 95% CI 0.41–1.0, *p* = 0.054 (Table 1). Similar results were obtained by analysis restricted to SS and Sβ^0^ patients.

## DISCUSSION

We examined the relationship between serum EPO concentration and thromboembolism in 204 UIC patients who had occurrence of venous thromboembolism recorded during a median follow-up of 6.4 years and 530 Walk-PHaSST patients with self-reported history of pulmonary embolism at baseline. In Walk-PHaSST which had centrally assayed EPO concentrations, we examined clinical covariates of EPO concentration and assessed genetic association of three fetal haemoglobin loci with EPO concentration, including *HBS1L-MYB* locus known to affect EPO levels in normal populations. Combined with PBMC gene expression data from 159 UIC patients, our analyses in both UIC and Walk-PHaSST pointed to erythroid expression variation of *EPOR* as a potential SCD-specific modulator of serum EPO concentration and thromboembolism risk. A major limitation of our study is its observational nature, where unmeasured confounders cannot be controlled for and causal relationship between circulating EPO levels and thromboembolism cannot be established. This causality cannot be rigorously deduced from the genetic associations of *EPOR* eQTL with EPO concentration and thromboembolism. Prospective multisite studies will be needed to validate these findings. Furthermore, future studies need to characterize all forms of thromboembolism, including arterial thromboembolism commonly presented as cerebrovascular accidents in SCD,^21^ for a more comprehensive understanding of EPO association with thrombotic complications.

Haemoglobin concentration and percent haemoglobin F are two clinical variables primarily correlated with serum EPO concentration in Walk-PHaSST and also in UIC (data not shown). More erythrocyte haemoglobin F causes less oxygen release to tissues due to high O_2_ affinity of haemoglobin F^15^ and hence a tendency to increase EPO synthesis by renal juxtaglomerular cells. At the same time, haemoglobin F interferes with deoxyHbS polymerization and alleviates haemolysis, reflected as higher haemoglobin concentration^35^ which in turn may lead to decreased EPO synthesis. Although the covariates we identified through the cross-sectional analysis (Figure 1) are lined with reasonable mechanisms for EPO variation, their causal relationship with EPO cannot be inferred from the observational data.

We identified an *EPOR* eQTL containing rs322139, the G allele of which associated with higher *EPOR* gene expression and lower serum EPO concentration. In differentiation of human erythroid progenitors in vitro, higher *EPOR* gene expression correlates with more cell surface EPOR.^36^ Higher *EPOR* gene expression in SCD erythroid progenitors may increase cell surface density of EPOR and erythropoiesis for a given level of EPO, which would in turn ameliorate hypoxia and decrease EPO production. This physiologic feedback mechanism is implied in familial erythrocytosis caused by a truncated cytoplasmic terminal of EPOR, in which affected individuals have low serum EPO concentrations and hypersensitivity to these low EPO levels.^37^ In contrast, a stop-gain variant of *EPOR* led to low responsiveness to EPO and elevated serum EPO concentration.^38^ The *EPOR* eQTL containing rs322139 that we report here was also identified in whole blood samples of Genotype-Tissue Expression from the normal population.^39^ The role of *EPOR* gene expression variation in modulating serum EPO concentration, however, appears to be specific to SCD or conditions associated with stress erythropoiesis. Our finding warrants further functional investigations, for example, in *EPOR*-knockdown SCD mice using short hairpin RNA under an erythroid specific promoter,^40^ where the effect of suppressed *EPOR* expression on serum EPO concentration could be assessed. Causal regulatory variation linked to rs322139 may be mapped through screening method that couples CRISPR inhibition with single cell RNA sequencing^41^ or flow cytometry^42^ in human erythroid cell lines.

Our present study shows that elevated serum EPO concentration associates with thromboembolism in SCD. It is of note that the thrombotic events in the UIC patients were significantly related to central venous catheters, orthopaedic procedures and hospitalization,^21^ which may exacerbate prothrombotic conditions in SCD. Previous studies have reported that high levels of circulating EPO stimulate the production of large-sized, hyperreactive platelets in dogs,^43^ mice and rats^44^ and healthy human volunteers.^18^ Consistently, we observed that elevated serum EPO concentration correlated with mean platelet volume in SCD patients (Table S7). Erythroid progenitors are closely related to megakaryocyte progenitors in haematopoiesis. EPOR is expressed in 59% of megakaryocyte-committed precursors sorted from wild-type murine bone marrow cells.^45^ EPO administration to rats increased the mean megakaryocyte diameter within 48 h and increased megakaryocyte maturation in all stages.^46^ In this study, the possible protective effect from thromboembolisms of the G allele of rs322139 in an *EPOR* eQTL might be attributable to its effect in decreasing circulating EPO concentration, which may have decreased overall EPO-EPOR signalling in megakaryocyte progenitors, resulting in less production of reactive platelets and lower risk for pulmonary embolism.

In summary, our study indicates that elevated serum EPO concentration may be a risk factor for thromboembolism in SCD and reveals *EPOR* gene expression variation as a potential novel modulator of EPO concentration and thromboembolism in SCD. Potential clinical applications of our findings include using assayed serum EPO concentration to identify subgroup of patients at high risk for thromboembolism for preventative management. Our finding of the relationship between *EPOR* expression and circulating EPO levels may suggest EPOR signalling as a potential therapeutical target for ameliorating prothrombotic conditions in SCD, by investigating the effect of drugs that modulate downstream kinase activity of the EPO-EPOR signalling pathway.^47,48^

## Supplementary Material

Data S1. Model of serum EPO concentration in Walk-PHaSST

Tables 1-7

Additional supporting information can be found online in the Supporting Information section at the end of this article.

## Figures and Tables

**TABLE 1:** Serum EPO concentration and the G allele of the *EPOR* rs322139 eQTL associated with venous thromboembolism in UIC and of pulmonary embolism in Walk-PHaSST SCD patients.

		UIC			Walk-PHaSST			Combined	
Predictor	Diagnosis	OR (95% CI)	*p*-value	#case/*N*	OR (95% CI)	*p*-value	#case/*N*	OR (95% CI)	*p*-value
Serum EPO Concentration	SCD	1.6 (0.98–2.6)	0.058	53/204	1.6 (0.88–2.8)	0.16	26/530	1.6 (1.1–2.3)	0.016
	SS/Sβ^0^	1.5 (0.87–2.4)	0.15	42/164	2.2 (1.1–4.3)	0.037	16/405	1.7 (1.1–2.6)	0.012
	SCD^a^	2.2 (1.2–3.9)	0.0068	43/176	1.7 (0.86–3.2)	0.17	19/381	1.9 (1.3–3)	0.0029
	SS/Sβ^0a^	2.0 (1.1–3.8)	0.023	32/136	2.5 (1.2–5.4)	0.030	11/265	2.2 (1.4–3.6)	0.0013

G allele of rs322139 in an *EPOR* eQTL	SCD	0.70 (0.41–1.2)	0.18	51/198	0.52 (0.23–1.2)	0.13	23/467	0.64 (0.41–1)	0.054
	SS/Sβ^0^	0.56 (0.29–1.1)	0.067	41/160	0.65 (0.23–1.8)	0.44	14/352	0.58 (0.34–1)	0.052

*Note*: Combined odds ratio (OR) and *p*-value were estimated by inverse variance weighting. Bias reduction logistic regression was used to account for the small numbers of cases. Patients in Walk-PHaSST did not have peripheral thrombosis recorded.

Abbreviations: SCD, sickle cell disease; UIC, University of Illinois at Chicago.

a Patients without recent blood transfusion.

**TABLE 2:** Association of the G allele of rs322139 in the *EPOR* eQTL (frequency = 0.29, imputation *r*^2^ = 0.96) with *EPOR* gene expression level in UIC PBMCs and with serum EPO concentration in Walk-PHaSST and UIC patients.

Diagnosis	*EPOR* expression in UIC PBMCs			Serum EPO concentration in walk-PHaSST			Serum EPO concentration in UIC		
	*β* (s.e.)	*p*-value	*N*	*β* (s.e.)	*p*-value	*N*	*β* (s.e.)	*p*-value	*N*
SCD	0.055 (0.013)	2.0 × 10^−5^	159	−0.18 (0.048)	0.00029	470	−0.17 (0.077)	0.032	186
SS/Sβ^0^	0.047 (0.015)	0.0015	139	−0.16 (0.056)	0.0054	353	−0.19 (0.092)	0.037	150
SCD^a^	0.050 (0.012)	8.9 × 10^−5^	145	−0.23 (0.056)	6.4 × 10^−5^	327	−0.18 (0.075)	0.017	179
SS/Sβ^0a^	0.040 (0.014)	0.0060	126	−0.21 (0.069)	0.0030	222	−0.21 (0.090)	0.021	143

Abbreviations: PBMC, peripheral blood mononuclear cells; SCD, Sickle cell disease; s.e., standard error; UIC, University of Illinois at Chicago.

a Patients without recent blood transfusion.

## Data Availability

The data that support the findings of this study are available on request from the corresponding author. The data are not publicly available due to privacy or ethical restrictions.
