# Supplementary material for: Circulating erythropoietin concentration associates with thromboembolism in sickle cell disease
Source: Br J Haematol. Author manuscript; Available in PMC 2026 Jun 16. (PMC13266539; doi:10.1111/bjh.70517)
Supplement: Data S1. Model of serum EPO concentration in Walk-PHaSST [file NIHMS2179505-supplement-Data_S1__Model_of_serum_EPO_concentration_in_Walk-PHaSST.doc]

**Model of serum EPO concentration in Walk-PHaSST**

Patients currently receiving EPO therapy or with serum creatinine ≥2.0 mg/dL were excluded from analyses. Clinical variables examined included complete blood cell counts, hemoglobin fractionation, comprehensive metabolic panel, creatinine clearance, estimated glomerular filtration rate, serum concentrations of lactate dehydrogenase and ferritin, plasma concentration of b-type natriuretic peptide, electrolyte panel including calcium, magnesium, phosphorus, and carbon dioxide, hemolysis index,^1^ weight, height, body mass index, peripheral oxygen saturation, blood pressure test, and echocardiogram. To identify clinical variables correlating with EPO, serum EPO concentrations were first regressed on age, gender, clinical site, hemoglobin genotype, hydroxyurea treatment, blood transfusion within the past two months, and hemoglobin concentration in a multivariate model. Residuals were tested for Spearman’s correlation with the remaining clinical variables and an updated model was formed accruing the variable with the strongest correlation. The procedure was repeated until no remaining variable showed correlation with the residuals of the last updated model at P-value=0.05. Recent blood transfusion, clinical sites, hemoglobin genotype, and hydroxyurea were then sequentially removed from the model due to a lack of significance or strong correlation with hemoglobin concentration or percent hemoglobin F. The final model included hemoglobin concentration, percent hemoglobin F, creatinine clearance, serum ferritin concentration, peripheral oxygen saturation, and serum concentration of alanine transaminase.

Reference

1. Nouraie M, Lee JS, Zhang Y, et al. The relationship between the severity of hemolysis, clinical manifestations and risk of death in 415 patients with sickle cell anemia in the US and Europe*. Haematologi*ca. 2013;98(3):464-472.
